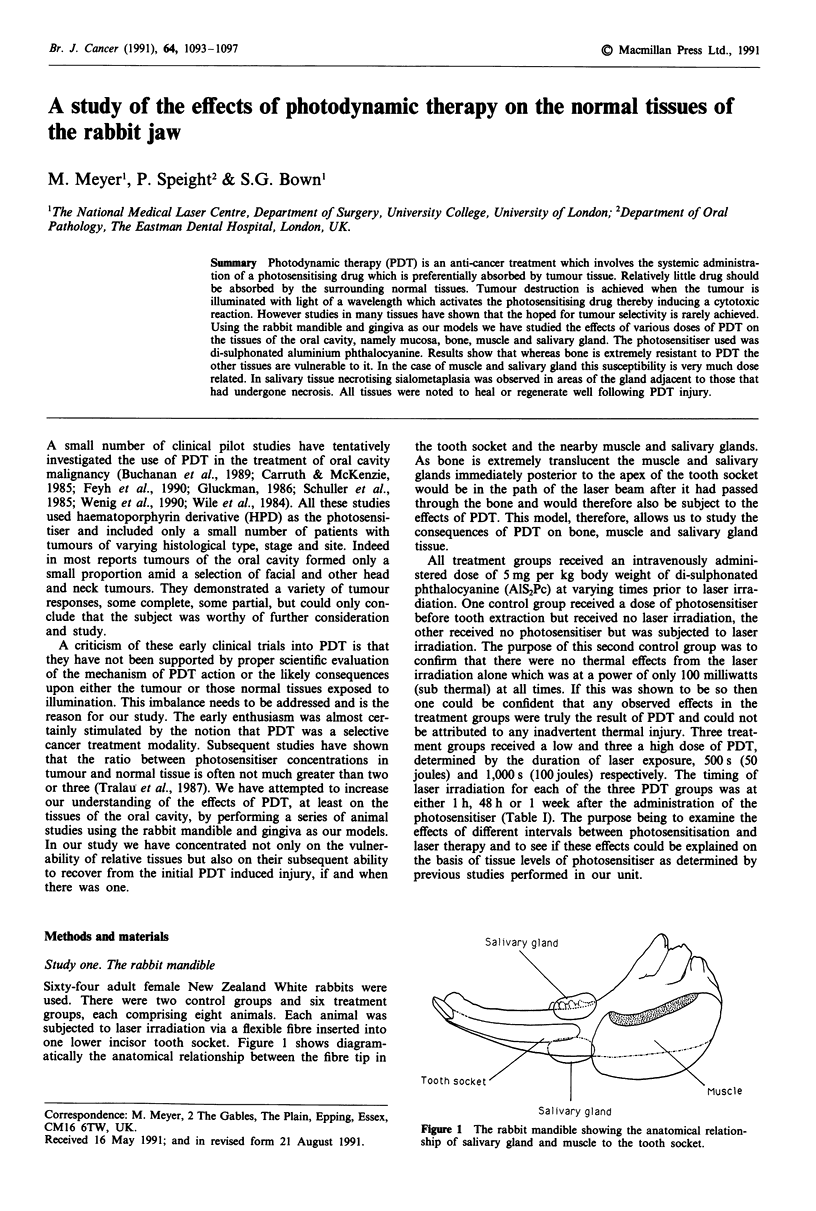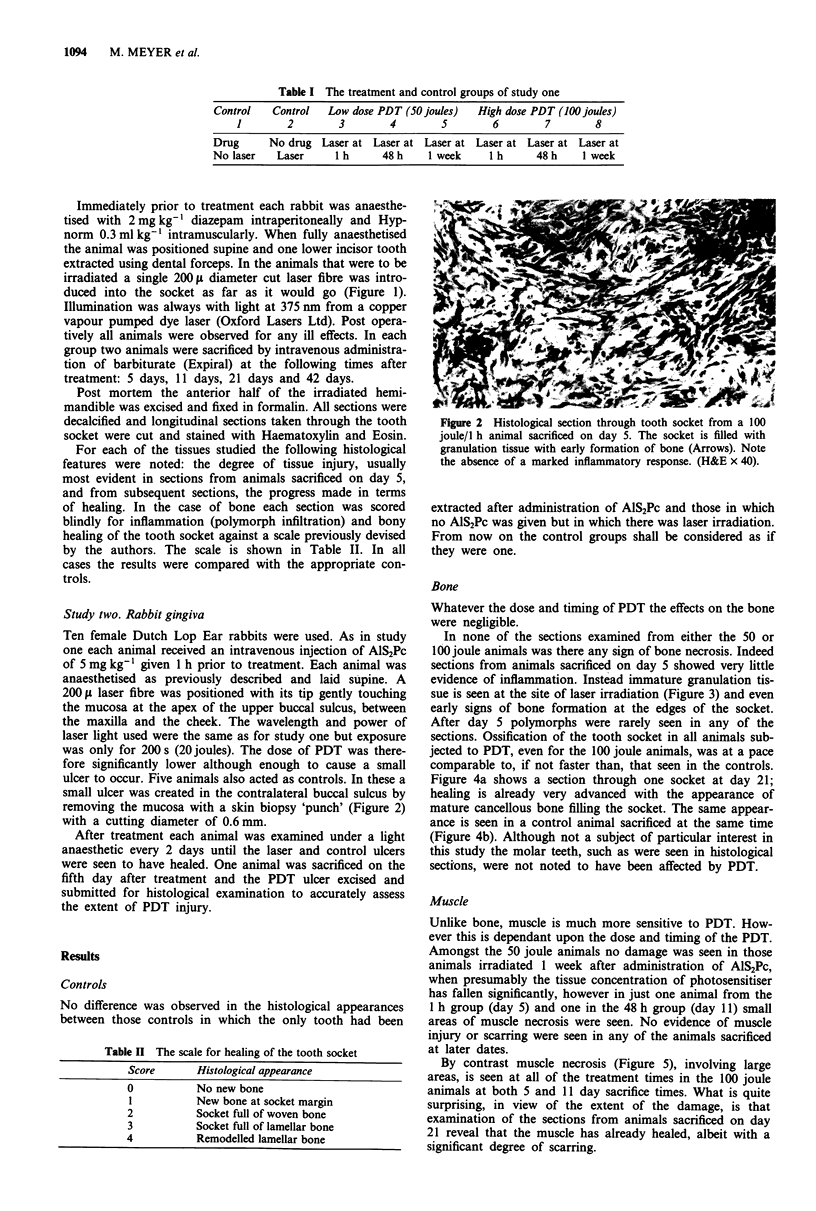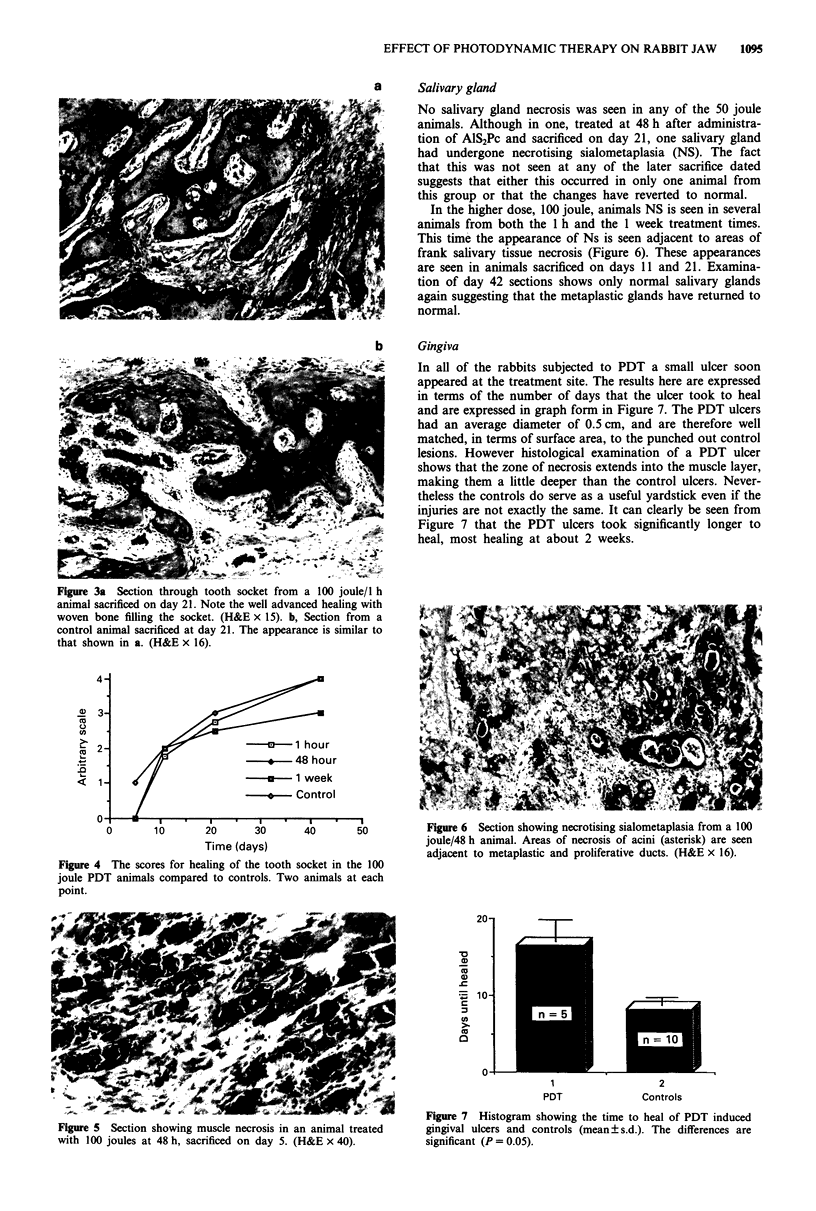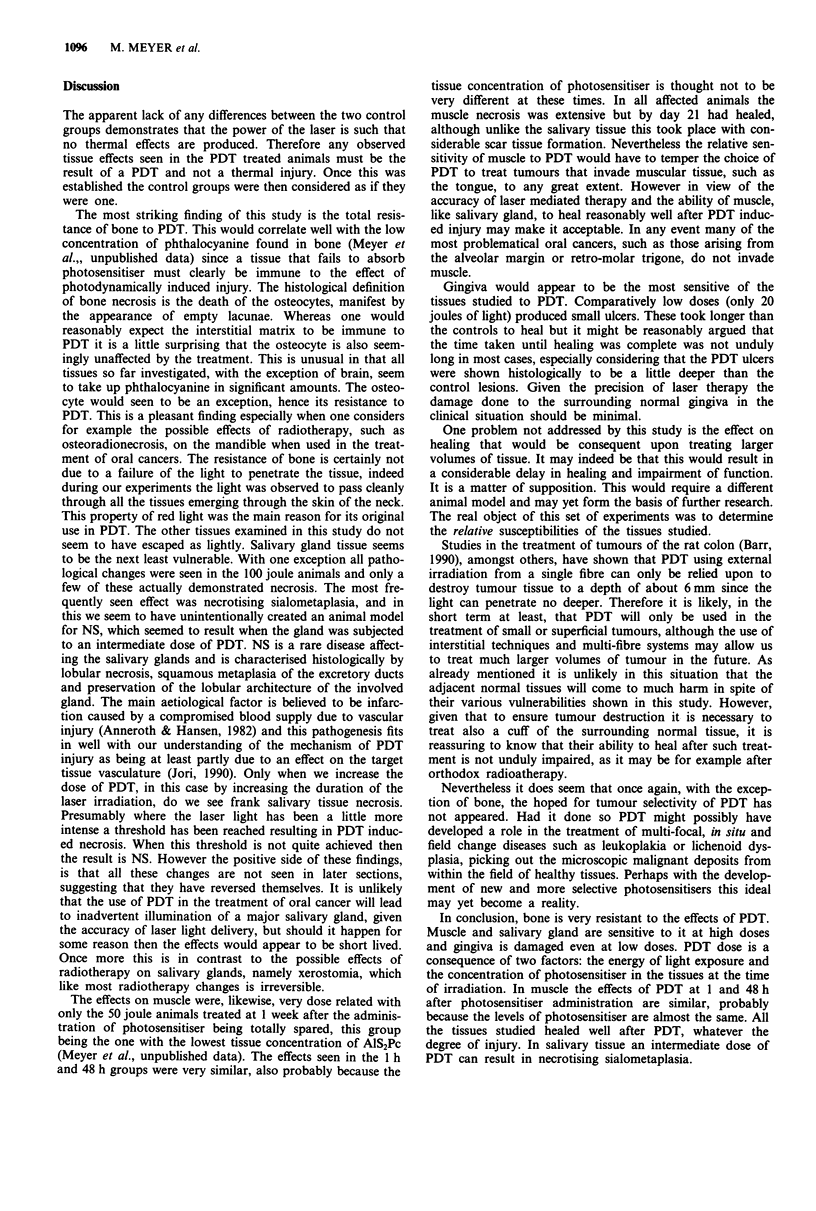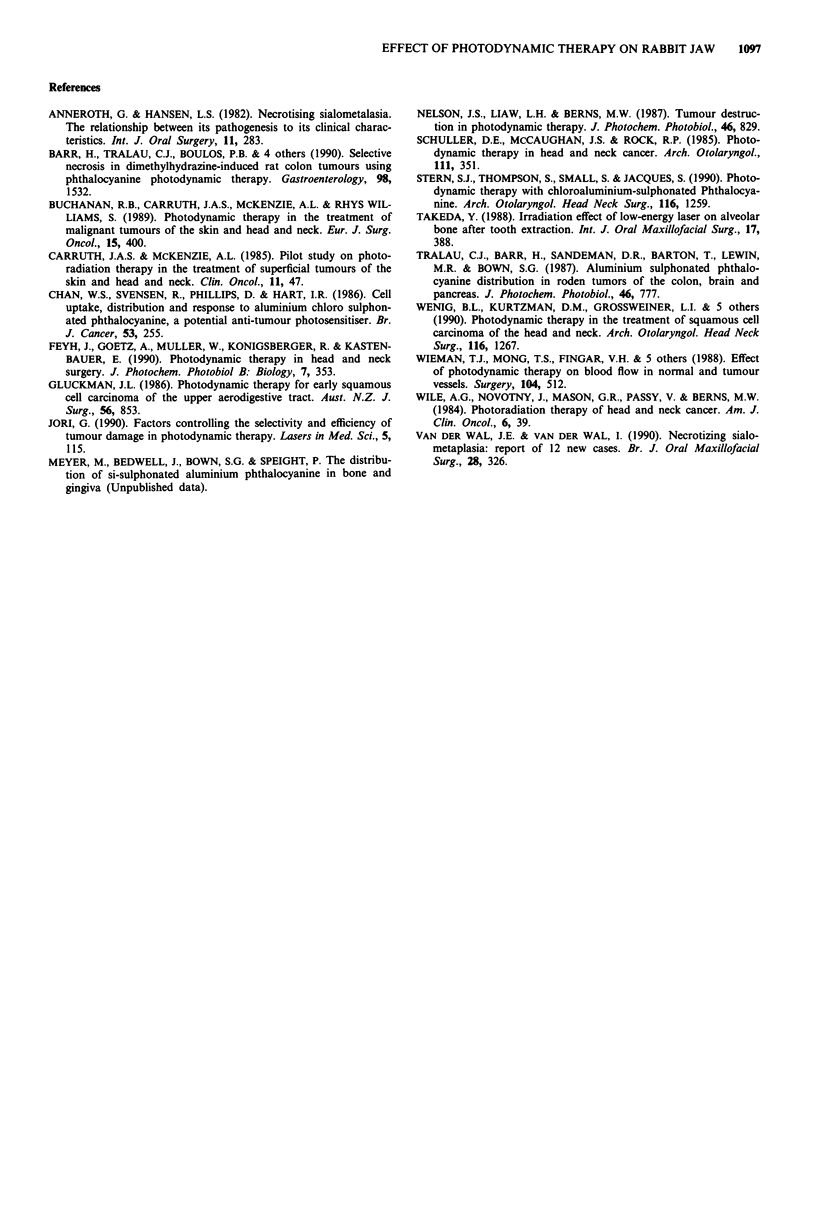# A study of the effects of photodynamic therapy on the normal tissues of the rabbit jaw.

**DOI:** 10.1038/bjc.1991.470

**Published:** 1991-12

**Authors:** M. Meyer, P. Speight, S. G. Bown

**Affiliations:** Department of Surgery, University College, University of London, UK.

## Abstract

**Images:**


*Br. J. Cancer* (1991), **64**, 1093–1097 © Macmillan Press Ltd., 1991

### A study of the effects of photodynamic therapy on the normal tissues of the rabbit jaw

M. Meyer[1], P. Speight[2] & S.G. Bown[1]

[1]*The National Medical Laser Centre, Department of Surgery, University College, University of London;* [2]*Department of Oral Pathology, The Eastman Dental Hospital, London, UK.*

**Summary** Photodynamic therapy (PDT) is an anti-cancer treatment which involves the systemic administration of a photosensitising drug which is preferentially absorbed by tumour tissue. Relatively little drug should be absorbed by the surrounding normal tissues. Tumour destruction is achieved when the tumour is illuminated with light of a wavelength which activates the photosensitising drug thereby inducing a cytotoxic reaction. However studies in many tissues have shown that the hoped for tumour selectivity is rarely achieved. Using the rabbit mandible and gingiva as our models we have studied the effects of various doses of PDT on the tissues of the oral cavity, namely mucosa, bone, muscle and salivary gland. The photosensitiser used was di-sulphonated aluminium phthalocyanine. Results show that whereas bone is extremely resistant to PDT the other tissues are vulnerable to it. In the case of muscle and salivary gland this susceptibility is very much dose related. In salivary tissue necrotising sialometaplasia was observed in areas of the gland adjacent to those that had undergone necrosis. All tissues were noted to heal or regenerate well following PDT injury.

A small number of clinical pilot studies have tentatively investigated the use of PDT in the treatment of oral cavity malignancy (Buchanan *et al.*, 1989; Carruth & McKenzie, 1985; Feyh *et al.*, 1990; Gluckman, 1986; Schuller *et al.*, 1985; Wenig *et al.*, 1990; Wile *et al.*, 1984). All these studies used haematoporphyrin derivative (HPD) as the photosensitiser and included only a small number of patients with tumours of varying histological type, stage and site. Indeed in most reports tumours of the oral cavity formed only a small proportion amid a selection of facial and other head and neck tumours. They demonstrated a variety of tumour responses, some complete, some partial, but could only conclude that the subject was worthy of further consideration and study.

A criticism of these early clinical trials into PDT is that they have not been supported by proper scientific evaluation of the mechanism of PDT action or the likely consequences upon either the tumour or those normal tissues exposed to illumination. This imbalance needs to be addressed and is the reason for our study. The early enthusiasm was almost certainly stimulated by the notion that PDT was a selective cancer treatment modality. Subsequent studies have shown that the ratio between photosensitiser concentrations in tumour and normal tissue is often not much greater than two or three (Tralau *et al.*, 1987). We have attempted to increase our understanding of the effects of PDT, at least on the tissues of the oral cavity, by performing a series of animal studies using the rabbit mandible and gingiva as our models. In our study we have concentrated not only on the vulnerability of relative tissues but also on their subsequent ability to recover from the initial PDT induced injury, if and when there was one.

### Methods and materials

### *Study one. The rabbit mandible*

Sixty-four adult female New Zealand White rabbits were used. There were two control groups and six treatment groups, each comprising eight animals. Each animal was subjected to laser irradiation via a flexible fibre inserted into one lower incisor tooth socket. Figure 1 shows diagramatically the anatomical relationship between the fibre tip in the tooth socket and the nearby muscle and salivary glands. As bone is extremely translucent the muscle and salivary glands immediately posterior to the apex of the tooth socket would be in the path of the laser beam after it had passed through the bone and would therefore also be subject to the effects of PDT. This model, therefore, allows us to study the consequences of PDT on bone, muscle and salivary gland tissue.

All treatment groups received an intravenously administered dose of 5 mg per kg body weight of di-sulphonated phthalocyanine ($AlS_2Pc$) at varying times prior to laser irradiation. One control group received a dose of photosensitiser before tooth extraction but received no laser irradiation, the other received no photosensitiser but was subjected to laser irradiation. The purpose of this second control group was to confirm that there were no thermal effects from the laser irradiation alone which was at a power of only 100 milliwatts (sub thermal) at all times. If this was shown to be so then one could be confident that any observed effects in the treatment groups were truly the result of PDT and could not be attributed to any inadvertent thermal injury. Three treatment groups received a low and three a high dose of PDT, determined by the duration of laser exposure, 500 s (50 joules) and 1,000 s (100 joules) respectively. The timing of laser irradiation for each of the three PDT groups was at either 1 h, 48 h or 1 week after the administration of the photosensitiser (Table I). The purpose being to examine the effects of different intervals between photosensitisation and laser therapy and to see if these effects could be explained on the basis of tissue levels of photosensitiser as determined by previous studies performed in our unit.

**Figure 1** The rabbit mandible showing the anatomical relationship of salivary gland and muscle to the tooth socket.

Correspondence: M. Meyer, 2 The Gables, The Plain, Epping, Essex, CM16 6TW, UK.
Received 16 May 1991; and in revised form 21 August 1991.

**Table I** The treatment and control groups of study one

| *Control* | *Control* | *Low dose PDT (50 joules)* | | | *High dose PDT (100 joules)* | | |
|---|---|---|---|---|---|---|---|
| *1* | *2* | *3* | *4* | *5* | *6* | *7* | *8* |
| Drug<br>No laser | No drug<br>Laser | Laser at<br>1 h | Laser at<br>48 h | Laser at<br>1 week | Laser at<br>1 h | Laser at<br>48 h | Laser at<br>1 week |

Immediately prior to treatment each rabbit was anaesthetised with 2 mg $kg^{-1}$ diazepam intraperitoneally and Hypnorm 0.3 ml $kg^{-1}$ intramuscularly. When fully anaesthetised the animal was positioned supine and one lower incisor tooth extracted using dental forceps. In the animals that were to be irradiated a single 200 μ diameter cut laser fibre was introduced into the socket as far as it would go (Figure 1). Illumination was always with light at 375 nm from a copper vapour pumped dye laser (Oxford Lasers Ltd). Post operatively all animals were observed for any ill effects. In each group two animals were sacrificed by intravenous administration of barbiturate (Expiral) at the following times after treatment: 5 days, 11 days, 21 days and 42 days.

Post mortem the anterior half of the irradiated hemimandible was excised and fixed in formalin. All sections were decalcified and longitudinal sections taken through the tooth socket were cut and stained with Haematoxylin and Eosin.

For each of the tissues studied the following histological features were noted: the degree of tissue injury, usually most evident in sections from animals sacrificed on day 5, and from subsequent sections, the progress made in terms of healing. In the case of bone each section was scored blindly for inflammation (polymorph infiltration) and bony healing of the tooth socket against a scale previously devised by the authors. The scale is shown in Table II. In all cases the results were compared with the appropriate controls.

### *Study two. Rabbit gingiva*

Ten female Dutch Lop Ear rabbits were used. As in study one each animal received an intravenous injection of $AlS_2Pc$ of 5 mg $kg^{-1}$ given 1 h prior to treatment. Each animal was anaesthetised as previously described and laid supine. A 200 μ laser fibre was positioned with its tip gently touching the mucosa at the apex of the upper buccal sulcus, between the maxilla and the cheek. The wavelength and power of laser light used were the same as for study one but exposure was only for 200 s (20 joules). The dose of PDT was therefore significantly lower although enough to cause a small ulcer to occur. Five animals also acted as controls. In these a small ulcer was created in the contralateral buccal sulcus by removing the mucosa with a skin biopsy 'punch' (Figure 2) with a cutting diameter of 0.6 mm.

After treatment each animal was examined under a light anaesthetic every 2 days until the laser and control ulcers were seen to have healed. One animal was sacrificed on the fifth day after treatment and the PDT ulcer excised and submitted for histological examination to accurately assess the extent of PDT injury.

### Results

### *Controls*

No difference was observed in the histological appearances between those controls in which the only tooth had been extracted after administration of $AlS_2Pc$ and those in which no $AlS_2Pc$ was given but in which there was laser irradiation. From now on the control groups shall be considered as if they were one.

**Table II** The scale for healing of the tooth socket

| *Score* | *Histological appearance* |
|---|---|
| 0 | No new bone |
| 1 | New bone at socket margin |
| 2 | Socket full of woven bone |
| 3 | Socket full of lamellar bone |
| 4 | Remodelled lamellar bone |

**Figure 2** Histological section through tooth socket from a 100 joule/1 h animal sacrificed on day 5. The socket is filled with granulation tissue with early formation of bone (Arrows). Note the absence of a marked inflammatory response. (H&E × 40).

### *Bone*

Whatever the dose and timing of PDT the effects on the bone were negligible.

In none of the sections examined from either the 50 or 100 joule animals was there any sign of bone necrosis. Indeed sections from animals sacrificed on day 5 showed very little evidence of inflammation. Instead immature granulation tissue is seen at the site of laser irradiation (Figure 3) and even early signs of bone formation at the edges of the socket. After day 5 polymorphs were rarely seen in any of the sections. Ossification of the tooth socket in all animals subjected to PDT, even for the 100 joule animals, was at a pace comparable to, if not faster than, that seen in the controls. Figure 4a shows a section through one socket at day 21; healing is already very advanced with the appearance of mature cancellous bone filling the socket. The same appearance is seen in a control animal sacrificed at the same time (Figure 4b). Although not a subject of particular interest in this study the molar teeth, such as were seen in histological sections, were not noted to have been affected by PDT.

### *Muscle*

Unlike bone, muscle is much more sensitive to PDT. However this is dependant upon the dose and timing of the PDT. Amongst the 50 joule animals no damage was seen in those animals irradiated 1 week after administration of $AlS_2Pc$, when presumably the tissue concentration of photosensitiser has fallen significantly, however in just one animal from the 1 h group (day 5) and one in the 48 h group (day 11) small areas of muscle necrosis were seen. No evidence of muscle injury or scarring were seen in any of the animals sacrificed at later dates.

By contrast muscle necrosis (Figure 5), involving large areas, is seen at all of the treatment times in the 100 joule animals at both 5 and 11 day sacrifice times. What is quite surprising, in view of the extent of the damage, is that examination of the sections from animals sacrificed on day 21 reveal that the muscle has already healed, albeit with a significant degree of scarring.

**Figure 3a** Section through tooth socket from a 100 joule/1 h animal sacrificed on day 21. Note the well advanced healing with woven bone filling the socket. (H&E × 15). **b**, Section from a control animal sacrificed at day 21. The appearance is similar to that shown in **a**. (H&E × 16).

**Figure 4** The scores for healing of the tooth socket in the 100 joule PDT animals compared to controls. Two animals at each point.

**Figure 5** Section showing muscle necrosis in an animal treated with 100 joules at 48 h, sacrificed on day 5. (H&E × 40).

*Salivary gland*

No salivary gland necrosis was seen in any of the 50 joule animals. Although in one, treated at 48 h after administration of $AlS_2Pc$ and sacrificed on day 21, one salivary gland had undergone necrotising sialometaplasia (NS). The fact that this was not seen at any of the later sacrifice dated suggests that either this occurred in only one animal from this group or that the changes have reverted to normal.

In the higher dose, 100 joule, animals NS is seen in several animals from both the 1 h and the 1 week treatment times. This time the appearance of Ns is seen adjacent to areas of frank salivary tissue necrosis (Figure 6). These appearances are seen in animals sacrificed on days 11 and 21. Examination of day 42 sections shows only normal salivary glands again suggesting that the metaplastic glands have returned to normal.

*Gingiva*

In all of the rabbits subjected to PDT a small ulcer soon appeared at the treatment site. The results here are expressed in terms of the number of days that the ulcer took to heal and are expressed in graph form in Figure 7. The PDT ulcers had an average diameter of 0.5 cm, and are therefore well matched, in terms of surface area, to the punched out control lesions. However histological examination of a PDT ulcer shows that the zone of necrosis extends into the muscle layer, making them a little deeper than the control ulcers. Nevertheless the controls do serve as a useful yardstick even if the injuries are not exactly the same. It can clearly be seen from Figure 7 that the PDT ulcers took significantly longer to heal, most healing at about 2 weeks.

**Figure 6** Section showing necrotising sialometaplasia from a 100 joule/48 h animal. Areas of necrosis of acini (asterisk) are seen adjacent to metaplastic and proliferative ducts. (H&E × 16).

**Figure 7** Histogram showing the time to heal of PDT induced gingival ulcers and controls (mean ± s.d.). The differences are significant ($P = 0.05$).

**Discussion**

The apparent lack of any differences between the two control groups demonstrates that the power of the laser is such that no thermal effects are produced. Therefore any observed tissue effects seen in the PDT treated animals must be the result of a PDT and not a thermal injury. Once this was established the control groups were then considered as if they were one.

The most striking finding of this study is the total resistance of bone to PDT. This would correlate well with the low concentration of phthalocyanine found in bone (Meyer *et al.*,, unpublished data) since a tissue that fails to absorb photosensitiser must clearly be immune to the effect of photodynamically induced injury. The histological definition of bone necrosis is the death of the osteocytes, manifest by the appearance of empty lacunae. Whereas one would reasonably expect the interstitial matrix to be immune to PDT it is a little surprising that the osteocyte is also seemingly unaffected by the treatment. This is unusual in that all tissues so far investigated, with the exception of brain, seem to take up phthalocyanine in significant amounts. The osteocyte would seen to be an exception, hence its resistance to PDT. This is a pleasant finding especially when one considers for example the possible effects of radiotherapy, such as osteoradionecrosis, on the mandible when used in the treatment of oral cancers. The resistance of bone is certainly not due to a failure of the light to penetrate the tissue, indeed during our experiments the light was observed to pass cleanly through all the tissues emerging through the skin of the neck. This property of red light was the main reason for its original use in PDT. The other tissues examined in this study do not seem to have escaped as lightly. Salivary gland tissue seems to be the next least vulnerable. With one exception all pathological changes were seen in the 100 joule animals and only a few of these actually demonstrated necrosis. The most frequently seen effect was necrotising sialometaplasia, and in this we seem to have unintentionally created an animal model for NS, which seemed to result when the gland was subjected to an intermediate dose of PDT. NS is a rare disease affecting the salivary glands and is characterised histologically by lobular necrosis, squamous metaplasia of the excretory ducts and preservation of the lobular architecture of the involved gland. The main aetiological factor is believed to be infarction caused by a compromised blood supply due to vascular injury (Anneroth & Hansen, 1982) and this pathogenesis fits in well with our understanding of the mechanism of PDT injury as being at least partly due to an effect on the target tissue vasculature (Jori, 1990). Only when we increase the dose of PDT, in this case by increasing the duration of the laser irradiation, do we see frank salivary tissue necrosis. Presumably where the laser light has been a little more intense a threshold has been reached resulting in PDT induced necrosis. When this threshold is not quite achieved then the result is NS. However the positive side of these findings, is that all these changes are not seen in later sections, suggesting that they have reversed themselves. It is unlikely that the use of PDT in the treatment of oral cancer will lead to inadvertent illumination of a major salivary gland, given the accuracy of laser light delivery, but should it happen for some reason then the effects would appear to be short lived. Once more this is in contrast to the possible effects of radiotherapy on salivary glands, namely xerostomia, which like most radiotherapy changes is irreversible.

The effects on muscle were, likewise, very dose related with only the 50 joule animals treated at 1 week after the administration of photosensitiser being totally spared, this group being the one with the lowest tissue concentration of $AlS_2Pc$ (Meyer *et al.*, unpublished data). The effects seen in the 1 h and 48 h groups were very similar, also probably because the tissue concentration of photosensitiser is thought not to be very different at these times. In all affected animals the muscle necrosis was extensive but by day 21 had healed, although unlike the salivary tissue this took place with considerable scar tissue formation. Nevertheless the relative sensitivity of muscle to PDT would have to temper the choice of PDT to treat tumours that invade muscular tissue, such as the tongue, to any great extent. However in view of the accuracy of laser mediated therapy and the ability of muscle, like salivary gland, to heal reasonably well after PDT induced injury may make it acceptable. In any event many of the most problematical oral cancers, such as those arising from the alveolar margin or retro-molar trigone, do not invade muscle.

Gingiva would appear to be the most sensitive of the tissues studied to PDT. Comparatively low doses (only 20 joules of light) produced small ulcers. These took longer than the controls to heal but it might be reasonably argued that the time taken until healing was complete was not unduly long in most cases, especially considering that the PDT ulcers were shown histologically to be a little deeper than the control lesions. Given the precision of laser therapy the damage done to the surrounding normal gingiva in the clinical situation should be minimal.

One problem not addressed by this study is the effect on healing that would be consequent upon treating larger volumes of tissue. It may indeed be that this would result in a considerable delay in healing and impairment of function. It is a matter of supposition. This would require a different animal model and may yet form the basis of further research. The real object of this set of experiments was to determine the *relative* susceptibilities of the tissues studied.

Studies in the treatment of tumours of the rat colon (Barr, 1990), amongst others, have shown that PDT using external irradiation from a single fibre can only be relied upon to destroy tumour tissue to a depth of about 6 mm since the light can penetrate no deeper. Therefore it is likely, in the short term at least, that PDT will only be used in the treatment of small or superficial tumours, although the use of interstitial techniques and multi-fibre systems may allow us to treat much larger volumes of tumour in the future. As already mentioned it is unlikely in this situation that the adjacent normal tissues will come to much harm in spite of their various vulnerabilities shown in this study. However, given that to ensure tumour destruction it is necessary to treat also a cuff of the surrounding normal tissue, it is reassuring to know that their ability to heal after such treatment is not unduly impaired, as it may be for example after orthodox radioatherapy.

Nevertheless it does seem that once again, with the exception of bone, the hoped for tumour selectivity of PDT has not appeared. Had it done so PDT might possibly have developed a role in the treatment of multi-focal, *in situ* and field change diseases such as leukoplakia or lichenoid dysplasia, picking out the microscopic malignant deposits from within the field of healthy tissues. Perhaps with the development of new and more selective photosensitisers this ideal may yet become a reality.

In conclusion, bone is very resistant to the effects of PDT. Muscle and salivary gland are sensitive to it at high doses and gingiva is damaged even at low doses. PDT dose is a consequence of two factors: the energy of light exposure and the concentration of photosensitiser in the tissues at the time of irradiation. In muscle the effects of PDT at 1 and 48 h after photosensitiser administration are similar, probably because the levels of photosensitiser are almost the same. All the tissues studied healed well after PDT, whatever the degree of injury. In salivary tissue an intermediate dose of PDT can result in necrotising sialometaplasia.

**References**

ANNEROTH, G. & HANSEN, L.S. (1982). Necrotising sialometalasia. The relationship between its pathogenesis to its clinical characteristics. *Int. J. Oral Surgery*, **11**, 283.

BARR, H., TRALAU, C.J., BOULOS, P.B. & 4 others (1990). Selective necrosis in dimethylhydrazine-induced rat colon tumours using phthalocyanine photodynamic therapy. *Gastroenterology*, **98**, 1532.

BUCHANAN, R.B., CARRUTH, J.A.S., MCKENZIE, A.L. & RHYS WILLIAMS, S. (1989). Photodynamic therapy in the treatment of malignant tumours of the skin and head and neck. *Eur. J. Surg. Oncol.*, **15**, 400.

CARRUTH, J.A.S. & MCKENZIE, A.L. (1985). Pilot study on photoradiation therapy in the treatment of superficial tumours of the skin and head and neck. *Clin. Oncol.*, **11**, 47.

CHAN, W.S., SVENSEN, R., PHILLIPS, D. & HART, I.R. (1986). Cell uptake, distribution and response to aluminium chloro sulphonated phthalocyanine, a potential anti-tumour photosensitiser. *Br. J. Cancer*, **53**, 255.

FEYH, J., GOETZ, A., MULLER, W., KONIGSBERGER, R. & KASTENBAUER, E. (1990). Photodynamic therapy in head and neck surgery. *J. Photochem. Photobiol B: Biology*, **7**, 353.

GLUCKMAN, J.L. (1986). Photodynamic therapy for early squamous cell carcinoma of the upper aerodigestive tract. *Aust. N.Z. J. Surg.*, **56**, 853.

JORI, G. (1990). Factors controlling the selectivity and efficiency of tumour damage in photodynamic therapy. *Lasers in Med. Sci.*, **5**, 115.

MEYER, M., BEDWELL, J., BOWN, S.G. & SPEIGHT, P. The distribution of si-sulphonated aluminium phthalocyanine in bone and gingiva (Unpublished data).

NELSON, J.S., LIAW, L.H. & BERNS, M.W. (1987). Tumour destruction in photodynamic therapy. *J. Photochem. Photobiol.*, **46**, 829.

SCHULLER, D.E., MCCAUGHAN, J.S. & ROCK, R.P. (1985). Photodynamic therapy in head and neck cancer. *Arch. Otolaryngol.*, **111**, 351.

STERN, S.J., THOMPSON, S., SMALL, S. & JACQUES, S. (1990). Photodynamic therapy with chloroaluminium-sulphonated Phthalocyanine. *Arch. Otolaryngol. Head Neck Surg.*, **116**, 1259.

TAKEDA, Y. (1988). Irradiation effect of low-energy laser on alveolar bone after tooth extraction. *Int. J. Oral Maxillofacial Surg.*, **17**, 388.

TRALAU, C.J., BARR, H., SANDEMAN, D.R., BARTON, T., LEWIN, M.R. & BOWN, S.G. (1987). Aluminium sulphonated phthalocyanine distribution in roden tumors of the colon, brain and pancreas. *J. Photochem. Photobiol.*, **46**, 777.

WENIG, B.L., KURTZMAN, D.M., GROSSWEINER, L.I. & 5 others (1990). Photodynamic therapy in the treatment of squamous cell carcinoma of the head and neck. *Arch. Otolaryngol. Head Neck Surg.*, **116**, 1267.

WIEMAN, T.J., MONG, T.S., FINGAR, V.H. & 5 others (1988). Effect of photodynamic therapy on blood flow in normal and tumour vessels. *Surgery*, **104**, 512.

WILE, A.G., NOVOTNY, J., MASON, G.R., PASSY, V. & BERNS, M.W. (1984). Photoradiation therapy of head and neck cancer. *Am. J. Clin. Oncol.*, **6**, 39.

VAN DER WAL, J.E. & VAN DER WAL, I. (1990). Necrotizing sialometaplasia: report of 12 new cases. *Br. J. Oral Maxillofacial Surg.*, **28**, 326.